# Does Training and Support of General Practitioners in Intensive Treatment of People with Screen-Detected Diabetes Improve Medication, Morbidity and Mortality in People with Clinically-Diagnosed Diabetes? Investigation of a Spill-Over Effect in a Cluster RCT

**DOI:** 10.1371/journal.pone.0170697

**Published:** 2017-02-02

**Authors:** Morten Charles, Mette V. Skriver, Simon J. Griffin, Rebecca K. Simmons, Daniel R. Witte, Else-Marie Dalsgaard, Torsten Lauritzen, Annelli Sandbæk

**Affiliations:** 1 Department of Public Health, Section of General Practice, Aarhus University, Denmark; 2 Department of Public Health, Section of Epidemiology, Aarhus University, Denmark; 3 Primary Care Unit, Institute of Public Health, University of Cambridge, Cambridge, United Kingdom; 4 MRC Epidemiology Unit, University of Cambridge School of Clinical Medicine, Cambridge, United Kingdom; 5 Danish Diabetes Academy, Odense University Hospital, Odense, Denmark; 6 Aarhus Institute of Advanced Studies, Høegh-Guldbergs Gade 6B, Aarhus C, Denmark; Florida International University Herbert Wertheim College of Medicine, UNITED STATES

## Abstract

**Introduction:**

Very few studies have examined the potential spill-over effect of a trial intervention in general practice. We investigated whether training and support of general practitioners in the intensive treatment of people with screen-detected diabetes improved rates of redeemed medication, morbidity and mortality in people with clinically-diagnosed diabetes.

**Methods:**

This is a secondary, post-hoc, register-based analysis linked to a cluster randomised trial. In the *ADDITION-Denmark* trial, 175 general practices were cluster randomised (i) to routine care, or (ii) to receive training and support in intensive multifactorial treatment of individuals with screen-detected diabetes (2001 to 2009). Using national registers we identified all individuals who were diagnosed with clinically incident diabetes in the same practices over the same time period. (Patients participating in the ADDITION trial were excluded). We compared rates of redeemed medication, a cardiovascular composite endpoint, and all-cause mortality between the routine care and intensive treatment groups.

**Results:**

In total, 4,107 individuals were diagnosed with clinically incident diabetes in *ADDITION-Denmark* practices between 2001 and 2009 (2,051 in the routine care group and 2,056 in the intensive treatment group). There were large and significant increases in the proportion of patients redeeming cardio-protective medication in both treatment groups during follow-up. After a median of seven years of follow-up, there was no difference in the incidence of a composite cardiovascular endpoint (HR 1.15, 95% CI 0.95 to 1.38) or all-cause mortality between the two groups (HR 1.08, 95% CI 0.94 to 1.23).

**Discussion:**

There was no evidence of a spill-over effect from an intervention promoting intensive treatment of people with screen-detected diabetes to those with clinically-diagnosed diabetes. Overall, the proportion of patients redeeming cardio-protective medication during follow-up was similar in both groups.

**Trial Registration:**

ClinicalTrials.gov NCT00237549

## Introduction

The benefit of managing cardiovascular risk factors among people with type 2 diabetes is well established [1]. However, effective implementation of evidence-based treatment is challenging. Studies suggest that many patients do not receive care according to current guidelines [2]. Changing health practitioner behaviour and their treatment schemes is not straightforward [3]. To improve the process of diabetes care, multifaceted professional interventions and organisational interventions that facilitate structured and regular review of patients have been shown to be effective in improving implementation of evidence-based treatment among clinicians [4].

In the Danish arm of the *ADDITION* trial, 175 general practices were cluster randomised to routine care or to receive training and support in the implementation of an intensive diabetes treatment program for individuals with screen-detected diabetes [5]. The ADDITION trial intervention was associated with a significant increase in redeemed cardio-protective medication and a non-significant 17% risk reduction in CVD events over five-years of follow-up [6]. Given the positive changes we managed to affect in health practitioner behaviour in the intensive treatment practices, we wanted to investigate whether the education and guidelines we offered them may also have had an impact on the management of patients with clinically-diagnosed diabetes. In the few studies which have examined the potential spill-over effect of a trial intervention in general practice, one found that no evidence for a spill-over effect in treatment from diabetes patients to those at high risk of developing diabetes [7], while another showed a positive change in practitioner behaviour in both control and intervention groups [8].

In order to assess a potential spill-over effect of the trial intervention among practices taking part in *ADDITION-Denmark*, we used national registry data to compare rates of redeemed cardio-protective medication, morbidity and mortality among people with clinically-diagnosed diabetes in the routine care and intensive treatment trial groups.

## Materials and Methods

### Study design–The ADDITION trial

*ADDITION-Denmark* consists of two phases: (i) a pragmatic screening programme, and (ii) a cluster-randomised parallel-group trial comparing the effects of intensive multifactorial treatment with routine care among individuals with screen-detected type 2 diabetes [5,6]. In brief, between 2001 and 2006, we performed a population-based stepwise screening programme among people aged 40 to 69 years without known diabetes in 175 general practices in Denmark. Participants diagnosed with type 2 diabetes were subsequently managed according to the treatment regimen to which their practice had been allocated: routine care or intensive treatment [9]. Ethical approval for the trial was granted by the Region Midt Ethical Committee, Denmark. This study was approved by the Danish Data Protection Agency and the Danish Health and Medicine Authority.

### Intervention

GPs and nurses received training and support in delivering intensive treatment via small group or practice-based educational meetings where treatment targets / algorithms, lifestyle advice, and supporting evidence were discussed. Patient audit and feedback was completed in face-to-face meetings held up to twice a year or was coordinated by post. Intensive treatment practices received additional funding to support the delivery of care, which included target-driven management of hyperglycaemia, blood pressure, and cholesterol levels by medical treatment and promotion of healthy lifestyles, based on the stepwise regimen used in the Steno-2 study and other trial results[6,9].

In the routine care group, general practitioners were advised to follow Danish national recommendations on diabetes treatment [10,11] and received no further follow-up.

### Study population

**Fig 1:**
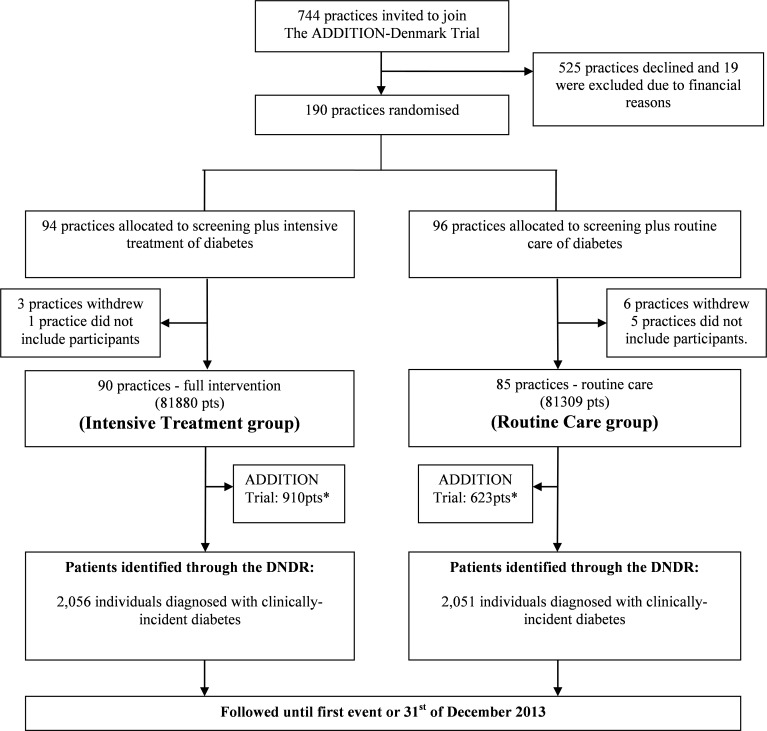
Flow of GP-Practices included in the Danish arm of the ADDITION trial and number of patients identified with clinically incident diabetes from the Danish National Diabetes Register (DNDR) during the intervention period of the ADDITION trial (2001–2009). *Patients with screen-detected diabetes included in the ADDITION Trial. Pts = patients.

This is a secondary, post-hoc, register-based analysis linked to the ADDITION-Denmark trial. In order to assess the potential spill-over effect of the intervention, we identified individuals who were diagnosed with clinically incident diabetes in the same 175 GP practices taking part in *ADDITION-Denmark* over the same time period as the intervention of the trial (2001 to 2009), using the target population from the *ADDITION-Denmark* (all patients between 40 and 69 years, n = 163,189). Our definition of clinically diagnosed diabetes was a proxy measure based on two different modes of identification. Participants were defined as having diabetes if they fulfilled at least one of the following criteria: (i) included on the Danish National Patient Register with a diagnosis of diabetes (ICD-8: 249, 250; ICD-10: E10-14, H36.0, O24, excluding O24.4); or (ii) redeemed glucose-lowering medication at least twice within six months during the study period (2001 to 2008). Patients with screen-detected diabetes who participated in the *ADDITION* trial were excluded. The practice and participants flow are shown in Fig 1.

We linked information about individuals with clinically incident diabetes to other Danish registers using unique civil registration numbers. We retrieved information on age, sex, education, marital status and redeemed cardio-protective medication (Danish National Prescription Registry[12]). Education was categorised according to Unesco’s International Standard Classification of Education^19^. We calculated the score for the Charlson Comorbidity Index[13] using information from the Danish National Patient Register from 10 years before inclusion in the study.

### Outcomes

Participants were followed for a median of 7.3 years to 31^st^ December 2013, when national registers were searched for information on vital status and incident CVD events. For death, the primary outcome was all-cause mortality (based on underlying cause of death). We used a composite of first event of cardiovascular death, non-fatal MI (ICD-10 codes I20 to I25, and I46) or non-fatal stroke (ICD-10 code I60-69, G45) to delineate an incident CVD event. Data on incident CVD events was gathered from the National Patient Registry, which records all in-patient and out-patient hospitalisations in Denmark.

### Statistical analysis

Baseline characteristics were summarised separately in the intensive treatment and routine care trial groups. Patient groups were compared using the χ2 test for categorical variables and the t test for continuous variables. P-values <0.05 were considered statistically significant. Date of entry to the study was date of diagnosis of clinically-incident diabetes. Individuals were censored on the date of first event, death, emigration or 31/12/2013, whichever was earliest. Hazard ratios comparing mortality and incident CVD events between the groups were estimated with a Cox proportional hazards regression model. We used a Nelson-Aalen hazard function to generate a cumulative incidenc curve based on this model. Since allocation to the treatment groups was at the practice level, robust standard errors were calculated that take into account the two-level structure of the data, using the “cluster()” option in Stata. We adjusted for age, sex and the Charlson Comorbidity Index Score. In a sensitivity analysis, we further adjusted for education level among those with complete data for this variable (118 people had missing data for education). We calculated the proportion of participants who redeemed prescriptions for six cardio-protective medication classes in both groups during the following times periods: 0 to 12 months before clinical diagnosis of diabetes, from 01/07/2008 to 30/06/2009, and from 01/01/2012 to 31/12/2012. All analyses were completed using Stata Version 14.1 (STATA Corp., College Station, Texas, USA).

## Results

**Table 1** shows the characteristics of individuals diagnosed with clinically-incident diabetes from *ADDITION-Denmark* practices between 2001 and 2009. The mean age (SD) of the cohort was 59.6 (8.2) years and 41% were female. Levels of education, marital status and the Charlson Comorbidity Index score were similar between the trial groups.

**Table 1:** Characteristics of individuals diagnosed with clinically-incident diabetes between 2001 and 2009 in *ADDITION-Denmark* general practices, by trial group.

	Intensive treatment trial group (n = 2,056)	Routine caretrial group (n = 2,051)	p-value
Female sex, n (%)	843 (41.0)	850 (41.4)	0.79
Age group (years), n (%)			
- 30–39	0 (0.)	3 (0.2)	0.29
- 40–49	301 (14.6)	4.6)	
- 50–59	728 (35.4)	4.5)	
- 60–69	801 (39.0)	0.8)	
- ≥70	226 (11.0)	206 (10.0)	
Mean age group (SD), years	59.6 (8.2)	59.6 (8.1)	1.00
Education level, n (%)			
- ≤ 10 years	892 (44.7)	850 (42.6)	0.31
- 10 to ≤ 15 years	828 (41.5)	3.9)	
- >15 years	275 (13.8)	269 (13.5)	
Married, n (%)	1,463 (76.0)	1,495 (77.8)	0.17
Charlson Comorbidity Index Score^a^, n (%)			
- 0	1,552 (75.5)	1,582 (77.1)	0.64
- 1	321 (15.6)	4.3)	
- 2	111 (5.4)	.3)	
- ≥3	72 (3.5)	67 (3.3)	
Year of clinically-incident diabetes diagnosis, n (%)			
- 2001–2002	221 (10.8)	222 (10.8)	0.33
- 2003–2004	576 (28.0)	8.4)	
- 2005–2006	595 (28.9)	6.5)	
- 2007–2008	664 (32.3)	703 (34.3)	

^a^ The Charlson Comorbidity Index Score included all 19 diseases except for diabetes. Patient groups were compared using the χ2 test for categorical variables and the t test for continuous variables.

Patterns of redeemed medication during follow-up are shown in Table 2. Before clinical diagnosis of diabetes, treatment groups redeemed similar proportions of anti-hypertensive, lipid-lowering, anti-depressant and anti-psychotic medication. However, the intensive treatment group redeemed higher levels of glucose-lowering and anti-thrombotic medication before diagnosis compared to the routine care group. During the follow-up period, there were large and statistically significant increases in the proportion of patients redeeming cardio-protective medication in both treatment groups. By the end of follow-up there were similar levels of cardio-protective medication in the two groups, although higher rates of redeemed anti-thrombotic medication persisted in the intensive treatment group. There were small increases in the proportion of the study population redeeming anti-depressant medication over time, and no change in the proportion receiving anti-psychotic medication.

**Table 2:** Rates of redeemed cardio-protective medication over time among individuals diagnosed with clinically-incident diabetes between 2001 and 2009 in *ADDITION-Denmark* general practices, by trial group.

	Intensive treatment trial group (n = 2,056)^1^	Routine care trial group (n = 2,051)^1^	p-value
	n (%)	n (%)	
**Anti-hypertensive medication**			
0–12 months before clinical diagnosis of diabetes	1,312 (63.8)	1,262 (61.5)	0.13
From 01/07/2008 to 30/06/2009	1,486 (78.3)	1,486 (78.9)	0.65
From 01/01/2012 to 31/12/2012	1,421 (83.6)	1,418 (82.1)	0.24
**Lipid-lowering medication**			
0–12 months before clinical diagnosis of diabetes	770 (37.5)	714 (34.8)	0.07
From 01/07/2008 to 30/06/2009	1,335 (71.3)	1,304 (68.7)	0.08
From 01/01/2012 to 31/12/2012	1,250 (73.6)	1,259 (72.9)	0.64
**Glucose-lowering medication**			
0–12 months before clinical diagnosis of diabetes	1,149 (55.9)	994 (48.5)	0.00*
From 01/07/2008 to 30/06/2009	1,416 (75.6)	1,385 (73.0)	0.07
From 01/01/2012 to 31/12/2012	1,318 (77.6)	1,350 (78.2)	0.67
**Anti-thrombotic medication**			
0–12 months before clinical diagnosis of diabetes	791 (38.5)	658 (32.1)	0.00*
From 01/07/2008 to 30/06/2009	1,129 (60.3)	1,002 (52.8)	0.00*
From 01/01/2012 to 31/12/2012	1,040 (61.2)	977 (56.6)	0.01*
**Anti-depressant medication**			
0–12 months before clinical diagnosis of diabetes	344 (16.7)	333 (16.2)	0.67
From 01/07/2008 to 30/06/2009	379 (20.2)	376 (19.8)	0.76
From 01/01/2012 to 31/12/2012	347 (20.4)	361 (20.9)	0.72
**Anti-psychotic medication**			
0–12 months before clinical diagnosis of diabetes	124 (6.0)	115 (5.6)	0.58
From 01/07/2008 to 30/06/2009	114 (6.1)	108 (5.7)	0.60
From 01/01/2012 to 31/12/2012	104 (6.1)	95 (5.5)	0.45

^1^At time of inclusion, thus numbers may not add up to total due to death or censoring. Patient groups were compared using the χ2 test for categorical variables and the t test for continuous variables.

* Statistically significant difference, P<0.05.

**Fig 2:**
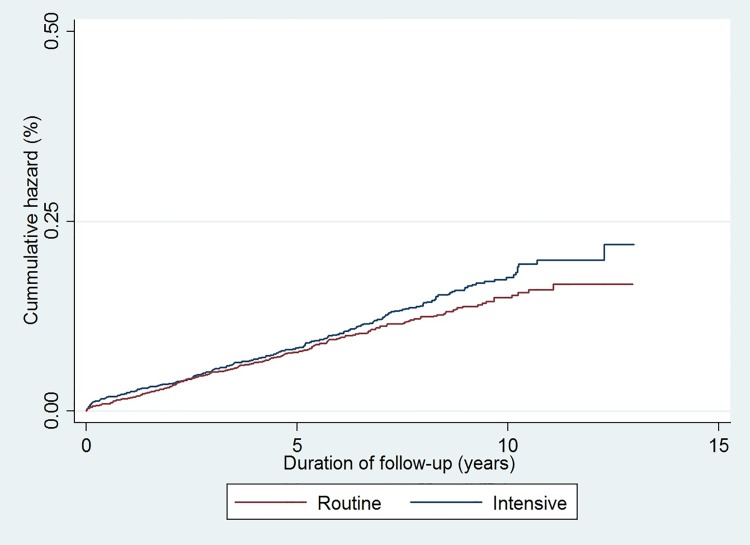
Cumulative incidence of a composite CVD endpoint (CVD death, non-fatal myocardial infarction or non-fatal stroke) among individuals diagnosed with clinically-incident diabetes from *ADDITION-Denmark* general practices by treatment groups from 2001–2012. (Blue line = Intensive treatment group, Red line = Routine care group).

The median (IQR) duration of follow-up was 7.3 years (5.7, 9.4). During follow-up, there were 289 cardiovascular events (14.1%) in the intensive treatment group (incidence (95% CI): 20.7 (18.4; 23.2 per 1,000 person-years) and 258 events (12.6%) in the routine care group (incidence (95% CI): 18.4 (16.3; 20.8) per 1,000 person-years) (**Table 3**). Following adjustment, there was no significant difference in incident CVD between the intensive treatment and routine care groups (HR 1.15,95% CI: 0.95 to 1.38) (**Fig 2**).

**Table 3:** Incidence of all-cause mortality and a composite cardiovascular event among individuals diagnosed with clinically-incident diabetes between 2001 and 2009 in *ADDITION-Denmark* general practices, by trial group.

	Intensive treatment trial group (n = 2,056)			Routine care trial group (n = 2,051)			Adjusted hazard ratios^1^ (95% CI)
	Number of events	Person-years of follow-up	Rate per 1,000pyrs (95%CI)	Number of events	Person-years follow-up	Rate per 1,000pyrs (95%CI)	
All-cause mortality	447	14,978	29.8 (27.2 to 32.7)	419	15,022	30.0 (27.2 to 33.0)	1.08 (0.94 to 1.23)
Composite cardiovascular event (first of CVD death, non-fatal IHD or non-fatal stroke)	289	13,959	20.7 (18.4 to 23.2)	258	14,044	18.4 (16.3 to 20.8)	1.15 (0.95 to 1.38)

^1^ Hazard ratios were estimated with a Cox proportional hazards regression model. Robust standard errors were calculated that take into account the two-level structure of the data and any potential correlation between individuals within practices. Models were adjusted for age, sex, and the Charlson Comorbidity Index Score.

**Fig 3:**
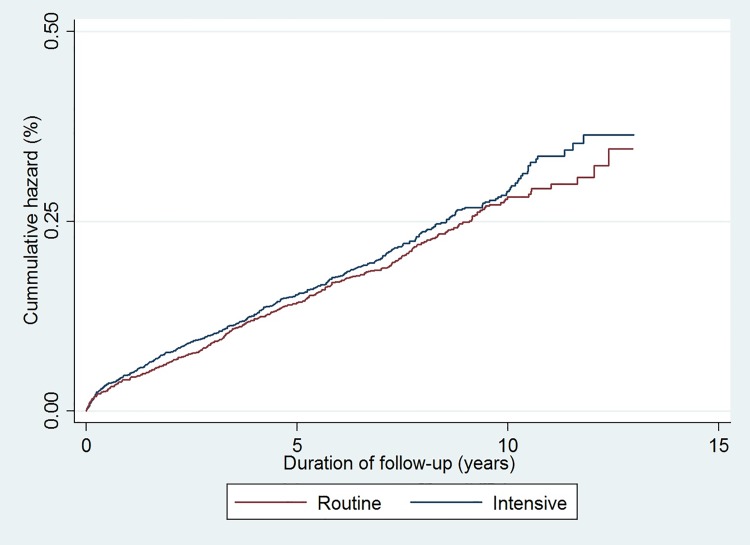
Cumulative incidence of all-cause mortality among individuals diagnosed with clinically-incident diabetes from *ADDITION-Denmark* general practices by the treatment group from 2001–2013. (Blue line = Intensive treatment group, Red line = Routine care group).

During follow-up, there were 447 deaths (21.7%) in the intensive treatment group (mortality rate (95% CI): 30.0 (27.2; 33.0) per 1,000 person-years) and 419 deaths (20.4%) in the routine care group (mortality rate (95% CI): 29.8 (27.2; 32.7) per 1,000 person-years). Following adjustment, there was no significant difference in all-cause mortality between the two groups (HR 1.08 (95% CI: 0.94 to 1.23)) (**Fig 3**). There was no difference in the overall result when adjusting for education level.

## Discussion

We found no evidence for a spill-over effect from a multi-faceted intervention promoting the intensive treatment of people with screen-detected diabetes to those with clinically-diagnosed diabetes. There was no difference in the risk of incident CVD and mortality between the intensive treatment and routine care groups during seven years of follow-up. Rates of cardio-protective medication increased significantly over time in both groups.

In a previous study examining the experience of individuals found with impaired fasting glycaemia or impaired glucose tolerance following screening in the *ADDITION-Denmark* trial, we showed that the training of general practitioners in intensive treatment of diabetes had no spill-over effect on these high-risk individuals[7]. There was no difference in rate of change in risk factor levels or lifestyle behaviours from screening to follow-up between individuals in the intensive treatment group compared to the routine group. There are very few other studies examining the spill-over effect of a trial intervention in general practice with which to compare our results. One study determining the effect of continuing medical education among family physicians on the quality of clinical care, found that when topics were of relatively great interest to physicians, the control group (who did not receive the continuing education package) showed as much improvement as the intervention group[8]. In terms of diabetes care, a recent meta-analysis of trials on quality improvement strategies concluded that interventions solely targeting health-care professionals seem to be beneficial only if baseline HbA_1c_ control is poor [14]. This finding was also observed in the *ADDITION-Denmark* trial [15]. This might be the case in this study as well. However, we do not have data on HbA1c values from the clinically-diagnosed individuals in our analysis to complete this sub-group analysis.

There are a further explanations for the lack of a spill-over effect observed in this trial. Firstly, we did not aim to affect change in health practitioner behaviour with regards to treatment of clinically incident diabetes patients. As such, training and support of general practitioners in the intensive treatment of people with screen-detected diabetes may not be associated with any benefits for individuals found with clinically-diagnosed diabetes in the same practices. An alternative explanation is that general practitioners in both trial groups improved their management of screen-detected and clinically diagnosed diabetes patients. GPs taking part in *ADDITION-Denmark* were self-selected and might have had a special interest in diabetes (28% of invited GPs took part in the trial). The trial also took place against a background of increasing national interest in screening and early treatment for diabetes, which might have encouraged practitioners to improve detection and management of CVD risk factors. Furthermore, we observed a large and significant increase in redeemed cardio-protective treatment in both trial groups during follow-up, suggesting that both intensive treatment and routine care GPs changed their prescribing behaviour among clinically-diagnosed patients. Both trial groups had room for improvement in GP prescribing e.g. only 78% of clinically-diagnosed individuals redeemed glucose-lowering medication at the end of follow-up. This finding mirrors the main trial results among screen-detected individuals, where both treatment groups received higher levels of cardio-protective medication by the end of follow-up and there were only modest differences in levels of treatment and cardiovascular risk factors at the five-year health examination[6].

### Strengths and limitations

The Danish registry system allows a unique investigation of the long-term experience of individuals diagnosed with clinically-incident diabetes in *ADDITION-Denmark* practices between 2001 and 2012. Trial groups were well balanced for patient level characteristics at baseline. Outcome ascertainment was very robust. The Danish National Death Registry estimates 100% coverage of mortality based on death certificates, while the National Patient Registry includes 99.4% of discharges from Danish hospitals. Deaths and incident CVD events were coded blind to study group.

Our definition of clinically diagnosed diabetes was a proxy measure based on date of inclusion in the Danish National Patient Register and redemption of glucose-lowering medication at least twice within six months during the study period (2001 to 2009). While we did not have formal clinical diagnosis of diabetes for each person included on the Register, a recent validation of the algorithm for including patients in the Register suggests that it has a sensitivity of ≥95% and a positive predictive value of around 80% [16]. A further advantage of using registry-defined diabetes is that the entire Danish population is covered by uniform inclusion criteria and the dropout rate is nil. Since patients selected for the *ADDITION-Denmark* were ≥40 years of age at the time of inclusion of GP practices, we assume that the number of type 1 diabetes cases is likely to be low and similar in both groups.

While we were able to compare trends in redeemed medication to explore a potential effect of the intervention on GP prescribing patterns, it would also have been useful to examine diet, physical activity and smoking behavior to examine potential effects on motivation to change lifestyle behaviour. However, these data are not available for individuals with clinically-diagnosed diabetes in *ADDITION-Denmark* practices.

In conclusion, there was no evidence of a spill-over effect from the intensive treatment of people with screen-detected diabetes to those with clinically-diagnosed diabetes. The risk of incident CVD and mortality did not differ between trial groups.

## References

[1] 2016) Standards of Medical Care in Diabetes-2016. Diabetes Care 39 Suppl 1: S1–S2. 39/Supplement_1/S1 [pii];.26696671

[2] Grol R, Grimshaw J (2003) From best evidence to best practice: effective implementation of change in patients' care. Lancet 362: 1225–1230. S0140-6736(03)14546-1 [pii];. 10.1016/S0140-6736(03)14546-1 14568747

[3] Cantillon P, Jones R (1999) Does continuing medical education in general practice make a difference? BMJ 318: 1276–1279. 10231265 10.1136/bmj.318.7193.1276 PMC1115655

[4] Grimshaw JM, Thomas RE, MacLennan G, Fraser C, Ramsay CR, Vale L, Whitty P, Eccles MP, Matowe L, Shirran L, Wensing M, Dijkstra R, Donaldson C (2004) Effectiveness and efficiency of guideline dissemination and implementation strategies. Health Technol Assess 8: iii–72. 94-08-29 [pii]. 10.3310/hta8060 14960256

[5] Lauritzen T, Griffin S, Borch-Johnsen K, Wareham NJ, Wolffenbuttel BH, Rutten G (2000) The ADDITION study: proposed trial of the cost-effectiveness of an intensive multifactorial intervention on morbidity and mortality among people with Type 2 diabetes detected by screening. Int J Obes Relat Metab Disord 24 Suppl 3: S6–11. 11063279 10.1038/sj.ijo.0801420

[6] Griffin SJ, Borch-Johnsen K, Davies MJ, Khunti K, Rutten GE, Sandbaek A, Sharp SJ, Simmons RK, van den Donk M, Wareham NJ, Lauritzen T (2011) Effect of early intensive multifactorial therapy on 5-year cardiovascular outcomes in individuals with type 2 diabetes detected by screening (ADDITION-Europe): a cluster-randomised trial. Lancet 378: 156–167. S0140-6736(11)60698-3 [pii];. 10.1016/S0140-6736(11)60698-3 21705063 PMC3136726

[7] Rasmussen SS, Glumer C, Sandbaek A, Lauritzen T, Borch-Johnsen K (2008) General effect on high-risk persons when general practitioners are trained in intensive treatment of type 2 diabetes. Scand J Prim Health Care 26: 166–173. 795457059 [pii];. 10.1080/02813430802264624 18677673 PMC3409605

[8] Sibley JC, Sackett DL, Neufeld V, Gerrard B, Rudnick KV, Fraser W (1982) A randomized trial of continuing medical education. N Engl J Med 306: 511–515. 10.1056/NEJM198203043060904 7057858

[9] Charles M, Ejskjaer N, Witte DR, Borch-Johnsen K, Lauritzen T, Sandbaek A (2011) Prevalence of Neuropathy and Peripheral Arterial Disease and the Impact of Treatment in People With Screen-Detected Type 2 Diabetes: The ADDITION-Denmark study. Diabetes Care 34: 2244–2249. dc11-0903 [pii];. 10.2337/dc11-0903 21816977 PMC3177734

[10] Royal College of General practitioners in Denmark (1999) Type 2 diabetes in general practice—Diagnosis and treatment.

[11] Royal College of General practitioners in Denmark. (2004) Type 2 diabetes in general practice—An evidence based guideline.

[12] Kildemoes HW, Sorensen HT, Hallas J (2011) The Danish National Prescription Registry. Scand J Public Health 39: 38–41. 39/7_suppl/38 [pii];. 10.1177/1403494810394717 21775349

[13] Charlson ME, Pompei P, Ales KL, MacKenzie CR (1987) A new method of classifying prognostic comorbidity in longitudinal studies: development and validation. J Chronic Dis 40: 373–383. 3558716 10.1016/0021-9681(87)90171-8

[14] Tricco AC, Ivers NM, Grimshaw JM, Moher D, Turner L, Galipeau J, Halperin I, Vachon B, Ramsay T, Manns B, Tonelli M, Shojania K (2012) Effectiveness of quality improvement strategies on the management of diabetes: a systematic review and meta-analysis. Lancet 379: 2252–2261. S0140-6736(12)60480-2 [pii];. 10.1016/S0140-6736(12)60480-2 22683130

[15] Lauritzen T, Sandbaek A, Carlsen AH, Borch-Johnsen K (2012) All-cause mortality and pharmacological treatment intensity following a high risk screening program for diabetes. A 6.6 year follow-up of the ADDITION study, Denmark. Prim Care Diabetes 6: 193–200. S1751-9918(12)00047-2 [pii];. 10.1016/j.pcd.2012.04.005 22595031

[16] Green A, Sortso C, Jensen PB, Emneus M (2015) Validation of the danish national diabetes register. Clin Epidemiol 7: 5–15. clep-7-005 [pii]. 10.2147/CLEP.S72768 25565889 PMC4274151

